# Limbus vertebrae of the cervical spine: A case report

**DOI:** 10.1002/ccr3.6567

**Published:** 2022-11-12

**Authors:** Eleni Pappa, Ioannis Chatzikomninos

**Affiliations:** ^1^ Spine and Scoliosis Department ”KAT” General Hospital of Athens Athens Greece

**Keywords:** cervical spine, limbus vertebrae

## Abstract

Limbus vertebra is a common radiological finding in an adult, especially in the mid‐lumbar region. However it is less commonly seen in the mid cervical region. A case of an anterior limbus vertebra seen on the cervical spine on an adult suffering from a T5 fracture is reported.

## Graphical abstract

The cervical anterior limbus vertebrae is an uncommon radiological finding in an adult. The orthopedic surgeons should be suspicious regarding that entity in the differential diagnosis of the radiological imaging of the cervical spine.

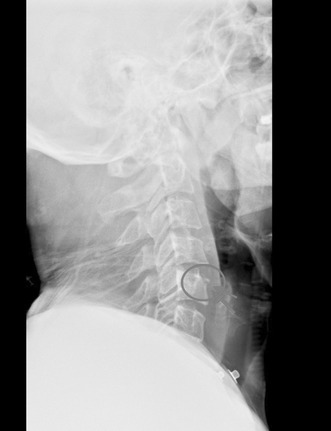

## 1 CASE REPORT

**FIGURE 1:**
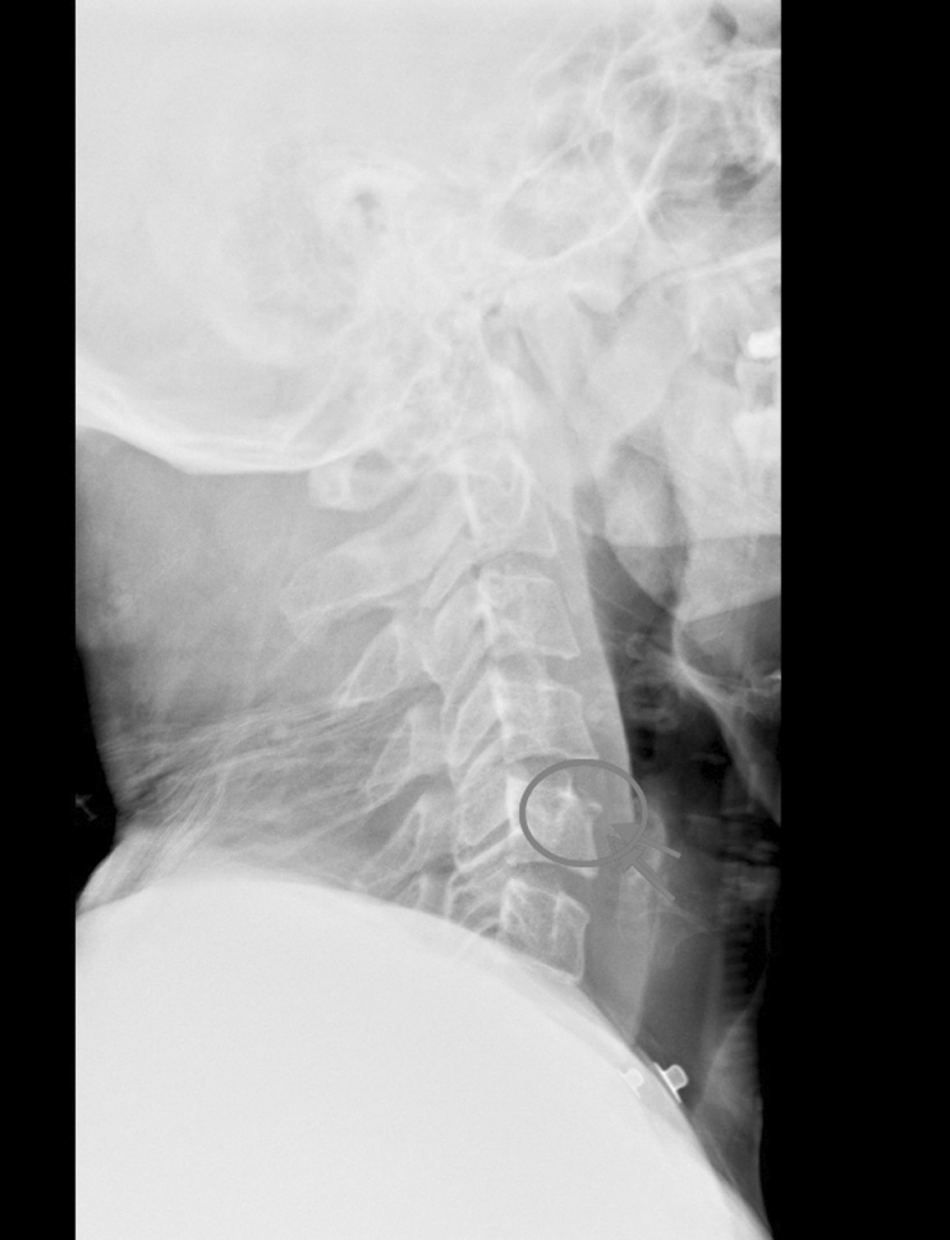
Lateral cervical spine X‐ray with the arrows and circle highlighting the limbus vertebra.

**FIGURE 2:**
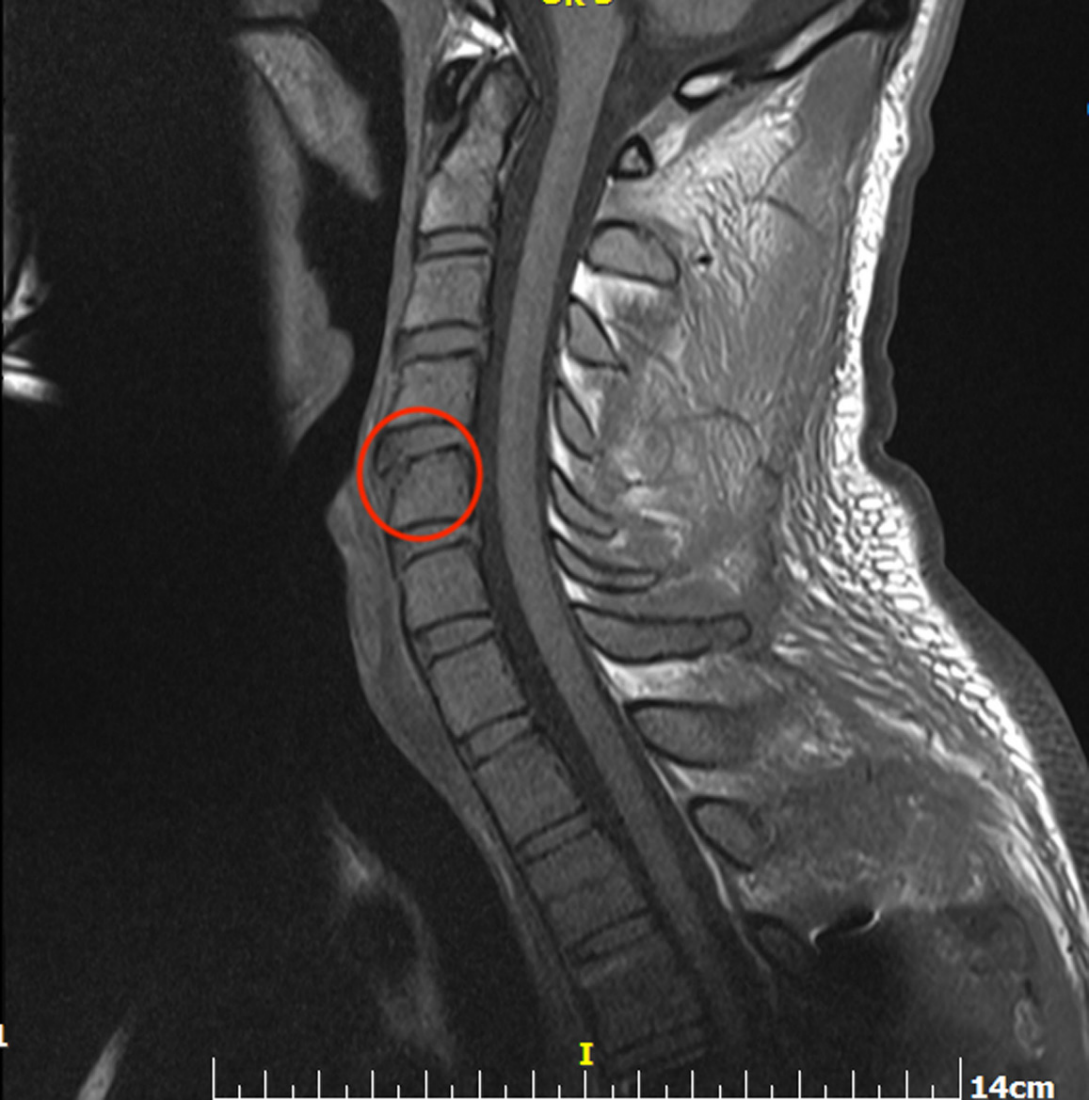
T1 magnetic resonance sequence of the cervical spine with the limbus vertebra encircled.

**FIGURE 3:**
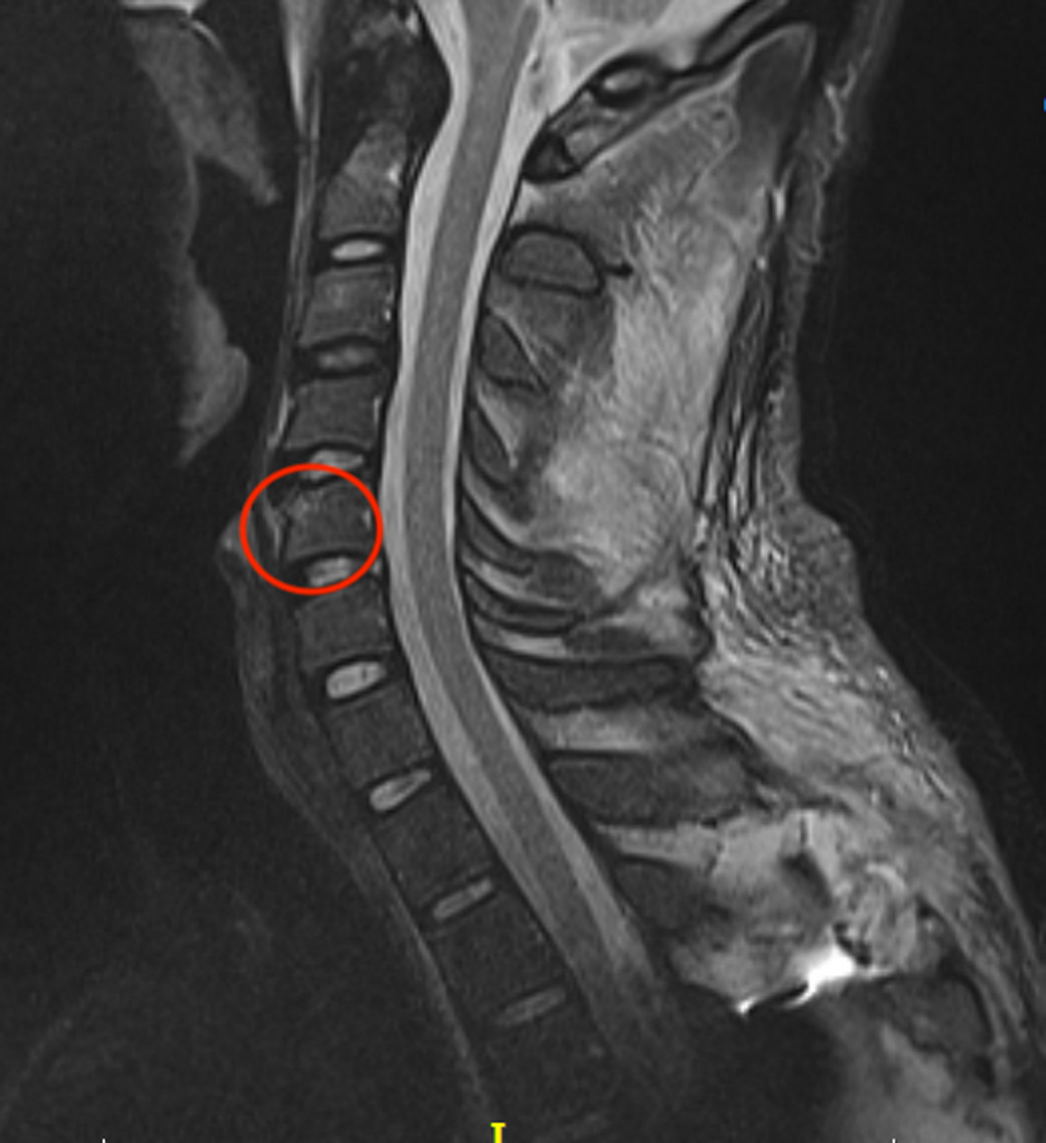
T2 magnetic resonance sequence of the cervical spine with the limbus vertebra encircled.

A 27‐year‐old male patient was admitted to our emergency department due to a motorcycle accident, suffering from a burst fracture of the T5 vertebrae. On the initial radiographic evaluation (Figure 1) of the cervical spine, there was a suspicion of a teardrop fracture of the C5 vertebrae, while the patient was asymptomatic. However, additional radiographic evaluation was carried out, where on the T1 (Figure 2) and T2 magnetic resonance sequence (Figure 3), the diagnosis of limbus vertebrae of the cervical spine was made; however, the differential diagnosis included teardrop vertebral fracture. The neurologic status of the patient was ASIA E and underwent posterior fusion of the thoracic spine on levels T3‐T8 without any neurologic impairment. A limbus vertebrae of the anterosuperior corner of a single vertebral body in the lumbar spine is the most common presentation, while it is quite uncommon on the cervical spine. Regarding the limbus vertebrae of the C5, the patient was treated conservatively.

Limbus vertebrae has been described for almost a century ago by Shmorl et al.; however, more light must be shed from the medical community regarding that entity, regarding the fact that its diagnosis may be challenging, especially in the cervical area where it is unusually present. Any anterosuperior triangular well‐corticated fragment of a vertebra especially in the midlumbar region, or in the cervical region as in our case, should be included in the differential diagnosis of limbus vertebrae by every orthopedic surgeon, including both CT and MRI evaluation.^1^, ^2^

## AUTHOR CONTRIBUTIONS

E.P drafted, gathered all the data information and wrote the manuscript. I.C reviewed and edited the manuscript.

## CONFLICT OF INTEREST

The authors have no conflicts of interest to declare.

## ETHICAL APPROVAL

Ethics approval was not required for this study based on local and national guidelines.

## CONSENT

Written informed consent was obtained from the patient to publish this report in accordance with the journal's patient consent policy.

## Data Availability

Research data are not shared.
